# Overexpression of *OsPIN2* Regulates Root Growth and Formation in Response to Phosphate Deficiency in Rice

**DOI:** 10.3390/ijms20205144

**Published:** 2019-10-17

**Authors:** Huwei Sun, Xiaoli Guo, Fugui Xu, Daxia Wu, Xuhong Zhang, Manman Lou, Feifei Luo, Guohua Xu, Yali Zhang

**Affiliations:** 1 Laboratory of Rice Biology in Henan Province, Collaborative Innovation Center of Henan Grain Crops, College of Agronomy, Henan Agricultural University, Zhengzhou 450002, China; guojingli188@126.com (X.G.); fugui_xu5683@126.com (F.X.); 2 State Key Laboratory of Crop Genetics and Germplasm Enhancement, Nanjing Agricultural University, Nanjing 210095, China; 2019203041@njau.edu.cn (D.W.); 2017103139@njau.edu.cn (X.Z.); 2018103147@njau.edu.cn (M.L.); 2018103148@njau.edu.cn (F.L.); ghxu@njau.edu.cn (G.X.); 3 Key Laboratory of Plant Nutrition and Fertilization in Low-Middle Reaches of the Yangtze River, Ministry of Agriculture, Nanjing Agricultural University, Nanjing 210095, China

**Keywords:** auxin, phosphate (P), OsPIN2, rice, root

## Abstract

The response of root architecture to phosphate (P) deficiency is critical in plant growth and development. Auxin is a key regulator of plant root growth in response to P deficiency, but the underlying mechanisms are unclear. In this study, phenotypic and genetic analyses were undertaken to explore the role of OsPIN2, an auxin efflux transporter, in regulating the growth and development of rice roots under normal nutrition condition (control) and low-phosphate condition (LP). Higher expression of *OsPIN2* was observed in rice plants under LP compared to the control. Meanwhile, the auxin levels of roots were increased under LP relative to control condition in wild-type (WT) plants. Compared to WT plants, two overexpression (OE) lines had higher auxin levels in the roots under control and LP. LP led to increased seminal roots (SRs) length and the root hairs (RHs) density, but decreased lateral roots (LRs) density in WT plants. However, overexpression of *OsPIN2* caused a loss of sensitivity in the root response to P deficiency. The OE lines had a shorter SR length, lower LR density, and greater RH density than WT plants under control. However, the LR and RH densities in the OE lines were similar to those in WT plants under LP. Compared to WT plants, overexpression of *OsPIN2* had a shorter root length through decreased root cell elongation under control and LP. Surprisingly, overexpression of *OsPIN2* might increase auxin distribution in epidermis of root, resulting in greater RH formation but less LR development in OE plants than in WT plants in the control condition but levels similar of these under LP. These results suggest that higher *OsPIN2* expression regulates rice root growth and development maybe by changing auxin distribution in roots under LP condition.

## 1. Introduction

Phosphorus (P) is essential nutrient for plant growth and development, and its deficiency dramatically affect crop productivity [1,2]. The changes of plant morphology in response to P- deficiency are an important mechanism for crops to optimize growth and productivity [3,4]. Increased root to shoot ratios and root surface areas induced by phosphate (P) deficiency have been reported in several plant species [5]. The changes in root morphology that occur under condition of P deficiency are complex and vary according to the experimental conditions and plant species. For example, a deficiency in P markedly inhibits primary root elongation in *Arabidopsis* [5,6]. This growth arrest is caused by reduced cell elongation and progressive cessation of cell proliferation in the root meristem that ultimately exhausts the primary root (PR) stem cell niche [7]. In contrast to *Arabidopsis*, in other plant species such as rice (*Oryza sativa* L.), elongation of the PRs occurs as a typical response to P deficiency [4,8]. Lateral root (LR) formation is also modulated under P deficiency in *Arabidopsis* and rice [9,10]. Significant P deficiency induces LR formation in *Arabidopsis* [7]. Conversely, in rice, the LR density decreases under P deficiency [10]. Meanwhile, the results of several studies have demonstrated root hair (RH) development and elongation in response to P deficiency [3,11,12]. Although the plasticity of root has been well documented in several plant species under P-deficiency. However, the mechanism in the regulatory cascade leading to change in root growth and formation are still not fully understood.

Besides environmental conditions, root growth and formation, are regulated by intrinsic factors such as plant hormones. Auxin is the key phytohormone to be discovered to regulate root growth in plants [4,11,13,14]. Auxin signaling is associated with changes in root morphology caused by P deprivation. As well, P-deprived were shown to undergo primary root growth arrest and show formation of LR with higher auxin levels in plants [3,15]. Most auxin is synthesised in aboveground tissues, such as shoot apices and young leaves, by *YUCCA* family genes [16,17,18] and then redistributed by auxin-influx carriers, such as AUX1/LAX family proteins, and auxin-efflux carriers, including ABCB/PGP and PIN family proteins [19,20,21]. 

Several studies have shown that polar auxin transport affects root growth [13,22,23,24,25]. For example, *OsPIN1* is involved in auxin-dependent adventitious root emergence in rice [13], and *Arabidopsis* mutants in *AtPIN2* display agravitropic root-growth phenotypes [22,23]. *OsPIN2* regulates root elongation and LR formation patterns via the modulation of auxin distribution in rice [25]. In addition, many studies have suggested that polar auxin transport regulates root growth and development in response to P supply [4,11]. In *Arabidopsis*, *OsAUX1* regulates auxin transport from the root apex to the differentiation zone, where this signal promotes root hair elongation when the roots encounter P deficiency [11]. In rice, *OsPIN1b* is involved in rice SR elongation by regulating root apical meristem activity in response to P deficiency [4]. PIN proteins are the major auxin efflux carriers in plants [19,26]. However, few studies have evaluated the role of *PINs* in regulating root growth and formation in response to low P condition.

Since auxin are involved in root growth modulated by P deficiency in several plant species. Further research is needed to understand possible links between auxin in the control of root development in response to low P condition. In this study, the role of *OsPIN2* in regulating rice root growth in response to P-limiting condition was examined using *OsPIN2* OE lines. Rice is an ideal model for auxin transport studies because of its small genome size and the availability of its complete genome sequence [27,28]. Upregulating expression of the *OsPIN2* gene in rice under condition of P deficiency revealed that the root growth patterns in these lines were related to elevated root auxin levels. In addition, comparison of the rice WT and OE lines carrying the auxin reporter construct *DR5::GUS*, following treatment with radiolabelled [^3^H] IAA and its polar transport, revealed that higher *OsPIN2* expression played important roles in regulating root growth and development by modifying auxin transport and distribution in roots under P deficiency.

## 2. Results

### 2.1. The Expression of OsPIN2 Was Induced by Low phosphate (LP) Supply

**Figure 1:**
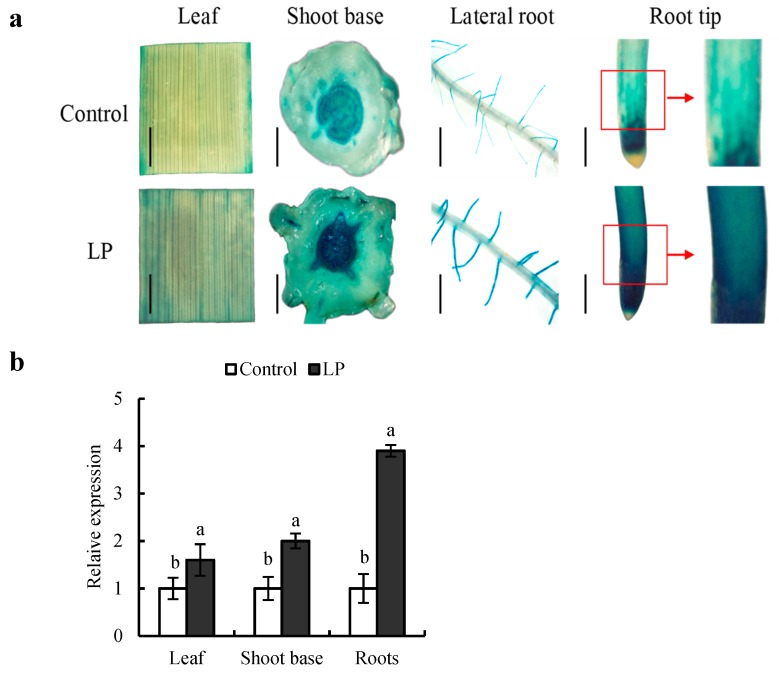
The expression of *OsPIN2* in rice plants. Seedlings were grown in hydroponic media containing normal nutrition (control, 300 μM P) or low P (LP, 10 µM) for 6 h. Bar = 1mm. (**a**) *pPIN2::GUS* expression in leaf, shoot base, lateral root and root tip. (**b**), *OsPIN2* expression in roots under control and LP conditions. *OsPIN2* expression was normalized to that of *OsACT*. h = hours. Data are means ± SE of three replicates and bars with different letters indicate significant differences in same organ (*p* < 0.05, ANOVA). All experiments included three independent biological replicates.

We evaluated the expression pattern of *OsPIN2* in rice plants using the GUS reporter gene and qRT-PCR in response to LP condition for 6 h. As shown in Figure 1a, *pPIN2::GUS* expression was highly expressed in shoot bases, lateral roots and root tips, but weakly in leaves, consistent with the previous results [14,29]. Compared with control treatment, *pPIN2::GUS* expression level in leaves, shoot bases and roots was increased by LP supply. As well, GUS location of *OsPIN2* in root tips were more concentrated in epidermis under LP relative to control condition (Figure 1a). The results of qRT-PCR showed the similar tendency as that of *pPIN2::GUS* expression (Figure 1b), suggesting the expression of *OsPIN2* gene was induced by LP supply.

### 2.2. Overexpression of OsPIN2 Regulates Auxin Levels in Roots

**Figure 2:**
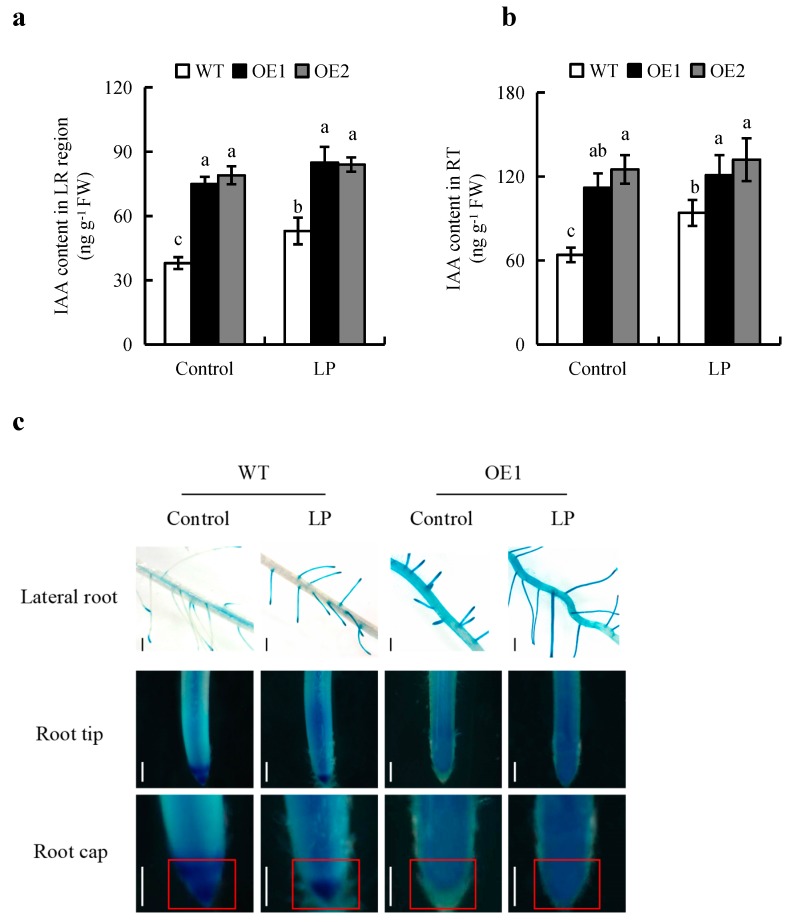
IAA content and histochemical localization of *DR5::GUS* in the wild type (WT, Nipponbare) and the overexpression of *OsPIN2* transgenic plants (OE1/OE2). Seedlings were grown in hydroponic media containing normal nutrition (control, 300 μM P) or low P (LP, 10 µM) for 7 d. (**a**,**b**), IAA concentration in lateral root (LR) (**a**) and root tip (RT) (**b**). (**c**), The expression of *DR5::GUS*. Bar = 1mm. d = days. Data are means ± SE and bars with different letters in the same root zone indicate significant difference at *p* < 0.05 tested with ANOVA. The red boxes indicate the root cap region. All experiments included three independent biological replicates.

As previously reported [25], the transcriptional levels o*f OsPIN2* were significantly increased in OE lines relative to WT plants (Figure S1). In WT plants, the IAA contents in LR regions and root tips (RTs, 0.5 cm) of plants under LP condition were significantly higher than those in plants grown in the control condition, by 65% and 47%, respectively (Figure 2a,b). Expression of the auxin-responsive reporter construct *DR5::GUS* was increased in the LRs and RTs in the LP condition, a pattern similar to that of IAA levels (Figure 2c). Compared to WT plants, the IAA contents in the LR regions and RTs of the OE lines in the control condition were significantly increased by 1-fold and 85%, respectively, and in the LP condition were significantly increased by 35% and 34%, respectively (Figure 2a,b). The expression results of the auxin-responsive reporter *DR5::GUS* and *GUS* enzyme activity were similar to the patterns of the IAA levels (Figure 2c and Figure S2). These results suggested that the root IAA levels were smaller differences between WT and OE lines grown in the LP condition. However, the *DR5::GUS* levels of root cap were decreased in OE lines and WT under LP relative to WT under control (Figure 2c). 

### 2.3. The Root Architectures of OE Lines Were Less Sensitive Than WT Plants to LP Condition

**Figure 3:**
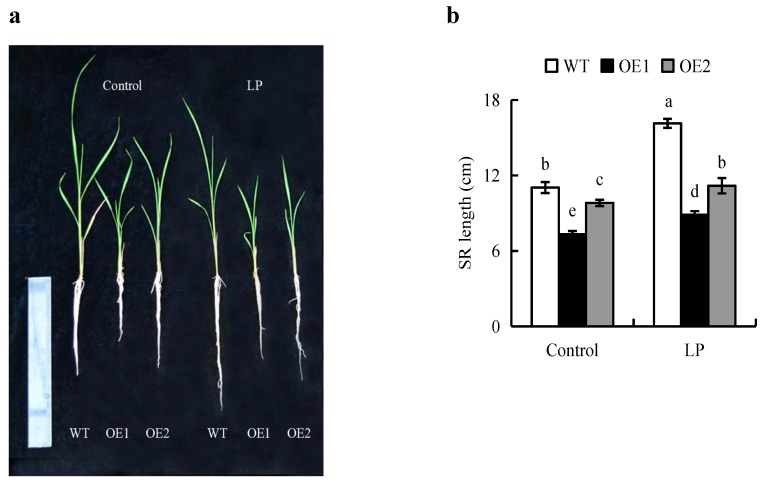
The morphology of the wild type (WT, Nipponbare) and the overexpression of *OsPIN2* transgenic plants (OE1/OE2). Seedlings were grown in hydroponic media containing normal nutrition (control, 300 μM P) or low P (LP, 10 µM). (**a**) The morphology of rice plants. (**b**) Seminal root (SR) length. Data are means ± SE and bars with different letters indicate significant differences in the same gene (*p* < 0.05, ANOVA). All experiments included three independent biological replicates.

**Figure 4:**
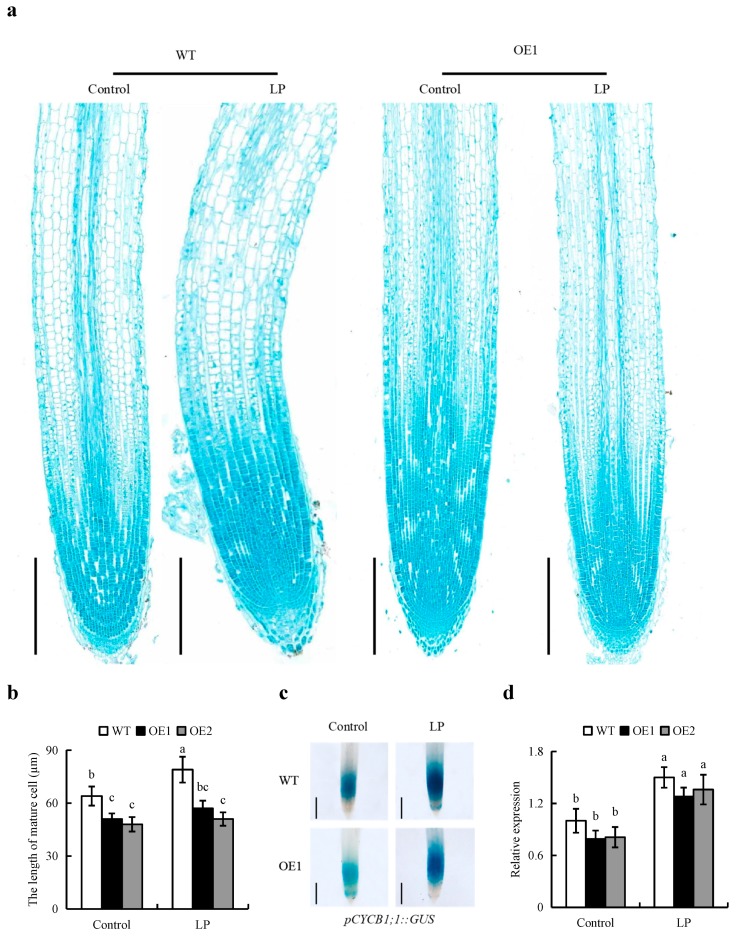
The epidermal cell lengths in the maturity zone and expression levels of *CYCB1;1* in the meristem zone in rice plants. Seedlings were grown in hydroponic medium containing normal nutrition (control, 300 μM P) and low P (LP, 10 µM) for 7 d. (**a**,**b**) Epidermal cell length of seminal root, Bar = 500 μm. (**c**) Cell cycle activity of the root meristem of seminal root, as monitored by the *pCYCB1;1::GUS* reporter. (**d**) The expression of *OsCYCB1;1* by qRT-PCR. Bar = 1mm. d = days. Data are means ± SE and bars with different letters indicate significant difference at *p* < 0.05 tested with ANOVA. All experiments included three independent biological replicates.

The experiment of root gravitropic was analyzed in Figure S3. The results indicated that overexpression of *OsPIN2* didn’t affect the gravitropic of root, consistent with the result of *OsPIN1b* overexpression lines [22]. As well, there was little effect of the NPA treatment on the root length of OE lines relative to WT plants (Figure S3). In contrast to WT plants, the root architectures of OE lines were less responsive to LP condition (Figure 3a). SR length of WT plants grown in LP solution increased by 46%, while that of the OE lines increased by approximately 14% (Figure 3b). To verify overexpression of *OsPIN2* inhibited root elongation, the length of epidermal cells in the maturity zone were analyzed in OE lines and WT plants (Figure 4a,b). The epidermal cells length in OE lines were shorter than that in WT both under control and LP conditions. The activity of *OsCYCB1;1::GUS* and the expression of *CYCB1;1* gene were stronger under P-deficiency compared to the control (Figure 4c). However, the similar levels of *pCYCB1;1::GUS* and *OsCYCB1;1* gene were observed in OE lines and WT plants under control and LP conditions.

**Figure 5:**
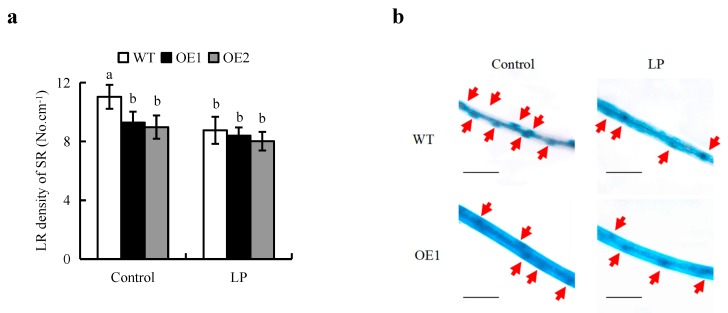
Lateral root (LR) density and lateral root (LR) primordia in wild type (WT, Nipponbare) and *OsPIN2* overexpression lines (OE1/OE2). Seedlings were grown in hydroponic media containing normal nutrition (control, 300 μM P) or low P (LP, 10 µM). (**a**) LR density of SR. (**b**) Lateral root (LR) primordia formation. Bar = 1mm. Data are means ± SE and bars with different letters indicate significant differences in the same gene (*p* < 0.05, ANOVA). The red arrow indicates the LR primordia. All experiments included three independent biological replicates.

LP corresponded to reductions in LR density of 21%, in the WT (Figure 5a). However, no differences in LR density were recorded in the OE lines between control and LP. The LR density in overexpression of *PIN2* was similar to that in WT under LP condition, the differences of roots were consistent with the result of IAA levels in roots. LR formation is dependent on LR primordia. The density of LR primordia significantly increased in WT under control relative to LP supply. As well, the LR primordia were decreased in OE lines relative to WT plants under control, but similar levels were found between WT and OE lines under LP condition (Figure 5b).

**Figure 6:**
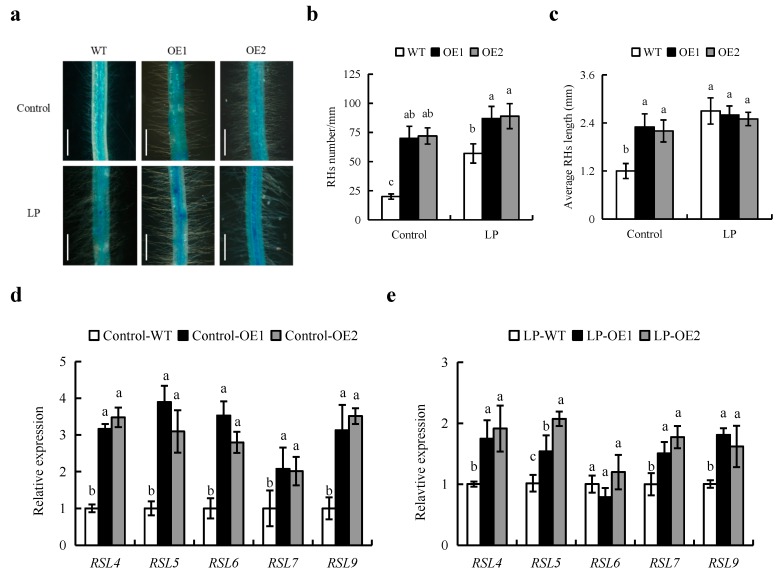
Root hairs (RHs) formation and qRT-PCR analysis of genes in wild type (WT, Nipponbare) and *OsPIN2* overexpression lines (OE1/OE2). Seedlings were grown in hydroponic media containing normal nutrition (control, 300 μM P) or low P (LP, 10 µM). (**a**) The morphology of RHs. (**b**) The density of RHs. (**c**) Average RHs length. Bar = 0.5mm. (**d**,**e**) The gene expression under normal nutrition (**d**) and low P; (**e**) for 6 h. Relative mRNA levels were normalised to *OsACT*. h = hours. Data are means ± SE and bars with different letters indicate significant differences in the same gene (*p* < 0.05, ANOVA). All experiments included three independent biological replicates.

The density and length of RH were significantly increased in *OsPIN2* overexpression relative to WT plants (Figure 6a–c). Under control treatment, RH density and average length were increased by 3.5- and 2- fold in OE plants relative to WT plants, respectively (Figure 6b,c). However, under LP treatment, in comparison to WT, only RH density were increased by 53% in OE plants. No differences were recorded in RH length between WT and OE lines (Figure 6b,c). These results suggested that overexpression of *OsPIN2* increased RH development in rice.

We monitored the expression levels of RH-formative genes in rice plants by qRT-PCR. The expression of *RSL4/5//7/9* were increased in OE lines relative to that in WT under control and LP condition (Figure 6d,e). The expression of *RSL6* genes was up-regulate in OE lines relative to WT under control but not under LP condition. 

### 2.4. Overexpressing OsPIN2 Regulates Root Development by Modifying Auxin Distribute

**Figure 7:**
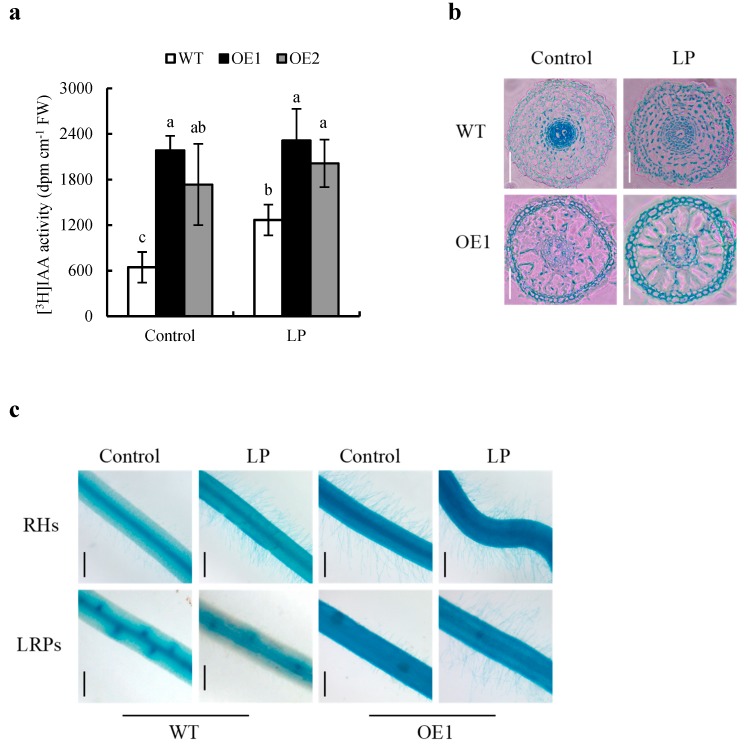
[^3^H]IAA transport and histochemical localization of *DR5::GUS* and root hair (RH) and lateral root primordia (LRP) formation. Seedlings were grown in hydroponic media containing normal nutrition (control; 2.5 mM N, 300 μM P) or low P (LP, 10 µM) for 7 d. (**a**) IAA transport for acropetal. (**b**) *DR5::GUS* distribution in transverse section. (**c**) RHs and lateral root primordia (LRPs) formation. d = days. Bar = 0.2 mm (**b**) and Bar = 0.5mm (**c**). Data are means ± SE and bars with different letters indicate significant differences in the same gene (*p* < 0.05, ANOVA). All experiments included three independent biological replicates.

The basipetal transport of auxin in WT plants was substantially increased in roots under the LP condition compared to the control condition (Figure 7a), suggesting that the basipetal transport of auxin was promoted by LP stress. In addition, the basipetal transport of auxin was significantly higher in the OE lines than in WT plants under both control and LP conditions. However, the basipetal transport of auxin were smaller differences between WT and OE lines under the LP condition than in the control condition (Figure 7a). 

To determine the reasons for the more RHs density in the OE lines, we further analyzed relative expression of auxin-responsive *DR5::GUS* reporter in transverse roots (1 mm from RT) of WT and OE lines, respectively. Higher *DR5::GUS* expressed in root epidermis of the OE lines relative to WT plants (Figure 7b,c) regardless of transverse section, suggesting overexpression of *OsPIN2* increased auxin distribution in epidermis of root.

## 3. Discussion

In this study, the level of auxin in WT plants was significantly greater in the LP condition than in the control condition. In addition, greater *OsPIN2* expression was involved in the LP-triggered induction of auxin transport changes in rice. The development of LRs and RHs in WT plants in the LP condition appeared similar to that in *OsPIN2*-overexpressing lines, suggesting that *OsPIN2* plays key roles in rice root growth and development in response to LP stress.

### 3.1. Overexpression of OsPIN2 Affects Root Elongation by Regulating the Elongation of Epidermal Cells that Leave the Root Meristem

The results of several studies have suggested that auxin is involved in root elongation under P deficiency [15]. Likewise, the auxin regulation of rice root elongation maybe related to its levels in root caps. Several studies have shown that both higher and lower auxin levels in root caps inhibit root growth [10,15], but that specific auxin levels in root caps promote root elongation [10,15]. For example, application of IAA was shown to increase auxin levels in root caps but reduce the root length in WT rice plants [15]. Rice plants grown in the presence of 1–1000 nM NAA (an exogenous auxin) was shown to decrease SR length with increasing NAA except at 1 nM, at which concentration a slight increase in SR length was found to occur [10]. Application of 20 µM NPA (a polar auxin transport inhibitor) to the root-shoot junction was shown to increase the root length in WT plants [25], but application of 300 nM NPA to roots reduced the auxin levels in the root caps and inhibited root elongation [4].

Several studies have suggested that *OsPIN2* affects root length by regulating auxin transport in the root cap [13,23,25]. In *Arabidopsis*, the *atpin2* mutant displays an agravitropic phenotype with reduced root elongation [23]. However, mutation of *OsPIN2* did not affect auxin transport or root length in rice [13]. It is possible that other *OsPIN* family genes compensate for the function of absent *OsPIN2*. *OsPIN2*-overexpressing plants have shown lower auxin accumulation in root caps than WT plants, and their root lengths change less in response to NPA, suggesting that these lines have a strong tolerance for NPA due to increased auxin transport [25]. In this study, in WT plants under the LP condition, the SR length was greater than that under the control condition, and the root cap auxin levels were lower (Figure 2c and Figure 3), consistent with the previous report [4,10]. These results suggest that LP condition cause a decrease in auxin accumulation in the root cap to levels that are ideal for the promotion of SR elongation. However, *OsPIN2*-overexpressing plants showed shorter SR lengths and lower auxin accumulation in root caps relative to those in WT plants in the LP condition (Figure 2c and Figure 3). These results indicate that overexpression of *OsPIN2* inhibit the elongation of SR maybe by reducing auxin accumulation in root caps (Figure 2c). 

Root length depends on two basal formation processes: Cell division in the RT meristem, and the final length of cells in the root maturity zone [30]. The activity of meristematic cells in the root meristem affects root elongation [31]. In rice, LP condition increase root meristem activity by regulating the expression of *OsCYCB1;1* [8]. In this study, compared to the control condition, the LP condition increased the *pCYCB1;1::GUS* and *OsCYCB1;1* expression levels in the RT and affected the length of mature cells (Figure 3a,b). However, the epidermal cell lengths in the OE lines were shorter than those in WT plants under both control and LP conditions. Similar *pCYCB1;1::GUS* and *OsCYCB1;1* expression levels were observed in the OE lines and WT plants under control and LP conditions (Figure 4c,d). These data show that *OsPIN2* overexpression affects root elongation primarily by affecting regulation of the elongation of epidermal cells that leave the root meristem.

### 3.2. Overexpression of OsPIN2 Regulate RHs and LRs Formation via Change Auxin Distribute in Root Transversal Section

RHs growth are regulated by auxin level and response in plants [11,15,32,33]. Our results showed that, when rice plants facing LP treatment, auxin response reporter *DR5::GUS* expression was significantly induced (Figure 7). As well, the changing tendency of auxin content under LP treatment paralleled *DR5::GUS* expression (Figure 7). Correspondingly, induced RH elongation and formation in WT plants under LP treatment were close linked to the auxin content and response. 

Increasing evidence found that auixn transport and response genes have been involved in RHs growth. *OsARF16*, an auxin response factor, promoted RHs elongation in rice [15]. Lower auxin response in roots and shorter RHs were recorded in *aux1* mutants under P deficiency [11]. Knockout of *AUX3* induced the RHs elongation relative to WT plants [14]. Above results suggested that the mechanisms of auxin influx carrier (AUX) regulated RHs elongation are complex. We found auxin efflux transporter, OsPIN2, participated mainly in RHs formation under control and LP treatments (Figure 6a–c).

Besides auxin, genetic factors play important roles in the development of RHs. *RSL Class II* family members are expressed in root epidermis and RH cells and as essential regulators of RH development in rice [34]. In this study, compared with WT, the expression of *RSL4/5/6/9* were up-regulated in OE lines under control and LP condition (Figure 6). These results suggested that *OsRSL* family genes play important roles in RHs deveplopment of OE lines. However, the transcriptional level of *OsRSL6* was increased in OE lines relative to WT plants under control condition but not under LP. This may be due to the expression of it was induced by LP condition in WT plants (Figure S4). These suggested the five RH-morphogenetic genes may be involved in RHs formation induced by OE plants.

*PIN2* plays a pivotal role in transmitting auxin through the peripheral layers of the root meristem and elongation zone [35]. In *Arabidopsis* and rice, *PIN2* localises to the basal side of cortical cells and to the apical sides of epidermal and root cap cells, and mainly regulates root basipetal auxin transport [14,36]. In this study, GUS location of *OsPIN2* in root tips were more concentrated in epidermis under LP relative to control condition (Figure 1a). The basipetal transport of auxin was significantly increased in OE lines relative to WT plants in both control and LP conditions (Figure 7a). Due to auxin responsive reporter *DR5::GUS* closely related to auxin content in plant root, just like discussed above, we investigated *DR5::GUS* expression level and found that overexpression of *OsPIN2* induced higher *DR5::GUS* expressed in epidermis cell in the OE lines relative to WT plants (Figure 7b). These suggested that overexpression of *OsPIN2* increased auxin distribution in epidermis of root. 

Auxin distribution in root transversal section affects LRs or RHs development [11,15,32,33,37]. The LR primordia (LRP) are formed from stele sheath cell [38]. However, the RHs are developed from epidermal cell [39]. Compared with WT, overexpression of *OsPIN2* increased RHs formation but decreased the LR formation under control treatment (Figure 7b,c). As well, these were smaller differences between WT and OE lines under LP condition relative to control condition (Figure 7b,c). These results suggested that higher expression of *OsPIN2* induced RHs development and reduced LRs formation probably by changing auxin distribution between stele and epidermis in rice roots.

## 4. Materials and Methods

### 4.1. Plant Materials

We obtained rice (*Oryza sativa* L.) of the Nipponbare ecotype for this study. Overexpression of the *OsPIN2* lines (OE1 and OE2) transgenic lines was performed following the previous methods [25,40].

### 4.2. Plant Growth

Plants were grown in pots in a greenhouse under natural light at day/night temperatures of 30/18 °C. We transplanted 1-week-old seedlings of uniform size and vigour into holes in a lid placed over the top of each pot. Nutrient solutions varying from one-quarter to full strength were applied for 1 week, followed by full-strength nutrient solution for another week. Pots receiving normal nutrition (control) were filled with and 300 µM P (Control, NaH_2_PO_4_), and those receiving P-deficient nutrition were filled with 10 µM P (LP). The full chemical composition of the International Rice Research Institute (IRRI) nutrient solution (pH 5.5) were 1.25 mM NH_4_NO_3_, 0.5 mM K_2_SO_4_, 1.0 mM CaCl_2_, 1.0 mM MgSO_4_·7H_2_O, 0.5 mM Na_2_SiO_3_, 20.0 µM Fe-EDTA, 9.0 µM MnCl_2_, 0.39 µM (NH_4_)_6_Mo_7_O_24_, 20.0 µM H_3_BO_3_, 0.77 µM ZnSO_4_, and 0.32 µM CuSO_4_. The nutrient solution was replaced with fresh solution daily. Each treatment consisted of four replicates arranged in a completely randomised design to avoid edge effects. All experiments included three independent biological replicates.

### 4.3. Measurement of Root System Architecture

The lengths of seminal roots (SRs) and root hairs (RHs), and the number of lateral roots (LRs), were chosen to study the effects of P on the root system. SR length was measured using a ruler. LR number of SR were calculated with eye. RH measurements were performed using a microscope (MVX10, Olympus) as described previously [41]. The number of RHs were counted on the RH zone of the SR. The LR primordia were observe by GUS (*DR5::GUS*) dye [37].

### 4.4. pPIN2::GUS Construct

The *pPIN2::GUS* fusion construct was transformed into rice plants. Histochemical localisation of *pPIN2::GUS* activity was performed at 30 min and 37 °C for the root and shoot base, and at 24 h and 37 °C for leaves. Stained tissues were photographed using a stereomicroscope (SZX2-ILLK, Olympus, Tokyo, Japan) and colour charge-coupled device (CCD) camera (Olympus) following the previous method [8,10]. Histochemical localisation was performed for histochemical GUS staining, and the results were photographed using the same microscope/CCD system.

### 4.5. Determination of IAA Content

Indole-3-acetic acid (IAA) concentrations in the shoot, shoot base, and root were determined as previously described [42]. After measuring fresh weight, each sample was immediately frozen in liquid N_2_. High-performance liquid chromatography (HPLC) was performed to measure free IAA concentration following the previous method [42]. A standard IAA sample was obtained from Sigma-Aldrich (Sigma, Louis, MO, USA).

The *pDR5::GUS* fusion construct was transformed into wild-type (WT) plants and overexpressed *OsPIN2* transgenic lines (OE1 and OE2) as previously described [25]. Plant issues were subjected to histochemical GUS staining analyses. Histochemical localisation of *DR5::GUS* activity was performed for the roots at 30 min and 37 °C. Stained tissues were photographed using the SZX2-ILLK microscope and colour CCD system described above.

GUS activity was examined according to previous method [43]. Samples were homogenised in GUS extraction buffer. Fluorometer values were compared with those of a 4-methylumbelliferone dilution series. Protein content was determined with a Bio-Rad protein assay kit using bovine serum albumin as the standard.

### 4.6. [^3^H]IAA Transport

Transport away (basipetal) was assayed in root segments 0–3 cm from the root tip. [^3^H]IAA solution (3 mL) was applied to the root tip placed horizontally on a plastic film. After incubation in a humid, dark environment for 18 h (overnight), root segments were the distal 1 cm from the root tip. [^3^H]IAA radioactivity was measured in the 2 cm long segments.

[^3^H]IAA polar transport was assayed as previous described [44]. Five replicate roots were sampled. The [^3^H]IAA solution contained 0.5 mM [^3^H]IAA (20 Ci mmol) in 2 % dimethylsulfoxide (DMSO), 25 mM MES (pH 5.2) and 0.25% agar.

### 4.7. Cortical Cell Length Analysis

The method of cortical cell length analysis as described previously [45]. The cortical cell visualized with a microscope (Olympus SZX16, Olympus) equipped with a colour CCD camera. The average cortical cell length of the maturation zone of seminal roots was obtained using a mixture of cortical cells with eight replicates in the maturation zone (on average per longitudinal section).

### 4.8. pCYCB1;1::GUS Construct

The *pCYCB1;1::GUS* fusion construct was transformed into wild-type (WT) plants and overexpressed *OsPIN2* transgenic lines (OE1 and OE2). Plants were stained for GUS activity in the root tips for 30 min at 37 °C. The root tips were subjected to histochemical GUS staining and photographed using a stereomicroscope (SZX2-ILLK, Olympus) equipped with a colour CCD camera.

### 4.9. Quantitative Reverse-Transcription Polymerase Chain Reaction Analyses

Total RNA was isolated from seedling roots, followed by RNA extraction, reverse transcription, and quantitative reverse-transcription polymerase chain reaction (qRT-PCR) analyses. Primer sets for the *RSLs* genes are listed in Table S1.

### 4.10. Data Analyses

Data were pooled for calculation of means and standard errors (SE) and subjected to one-way analysis of variance (ANOVA), followed by Ryan-Eynot-Gabriel-Welch F test at *p* < 0.05 to determine the statistical significance of differences between treatments. All statistical evaluations were conducted using SPSS (version 11.0) statistical software (SPSS Inc., Chicago, IL, USA). Different letters indicate significant differences (*p* < 0.05, ANOVA).

## 5. Conclusions

This study showed that overexpression of *OsPIN2* increases basipetal transport of auxin in roots and changes rice root morphology in response to P supply. And higher *OsPIN2* expression playes important roles in regulating root growth and development by modifying auxin transport and distribution in roots under P deficiency in rice.

## Supplementary Materials

Supplementary materials can be found at https://www.mdpi.com/1422-0067/20/20/5144/s1.
