# Supplementary material for: Overexpression of OsPIN2 Regulates Root Growth and Formation in Response to Phosphate Deficiency in Rice
Source: Int J Mol Sci. 2019 Oct 17;20(20):5144. doi: 10.3390/ijms20205144 (PMC6829224; doi:10.3390/ijms20205144)
Supplement: Supplementary file 1 [file ijms-20-05144-s001.pdf]

Supplementary

Table S1. The primers for qRT-PCR of *OsRSLs* and *OsPIN2* genes.

Gene	Primer sequence
OsRSL4	5'-CGTGCAAGAGGAAGGTTGAG-3'
	5'-CTTCTTTGACTGCGCGTTCT-3'
OsRSL5	5'-GTCGAATGGCAAGGCTCAAT-3'
	5'-CTTCACGTAATGCATTGCCTC-3'
OsRSL6	5'-CCTGCAGAACTTGGTTCCCA-3'
	5'-GCATACATCCACGTGTCATC-3'
OsRSL7	5'-CTCTATGCTAAGAGGAGAAG-3'
	5'-CATCCATGTGTCGTCAGAGC-3'
OsRSL9	5'-GGATCAATGAGAGGCTCAAG-3'
	5'-CGCAATTGGCGCATACATCCAC-3'
OsPIN2	5'-CAACACCTACTCCAGCCTC-3'
	5'-TGGACCAGTCAAGAACCTC-3'
OsActin	5'-CAACACCCCTGCTATGTACG-3'
	5'-CATCACCAGAGTCCAACACAA-3'

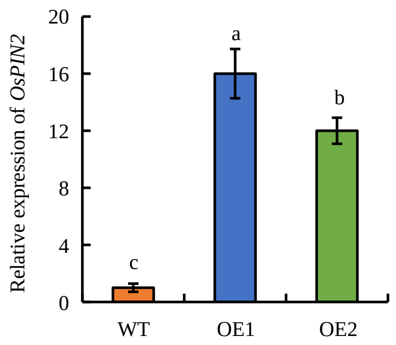

Figure S1. Transcriptional levels of *OsPIN2* in wild type (WT, Nipponbare) and *OsPIN2* overexpression lines (OE1/OE2). Seedlings were grown in hydroponic media containing normal nutrition (control, 300 μM P) for 7 d. Relative expression of *OsPIN2* were normalised to *OsACT* by qRT-PCR. d = days. Data are means ± SE and bars with different letters indicate significant differences in the same gene ($p < 0.05$, ANOVA).

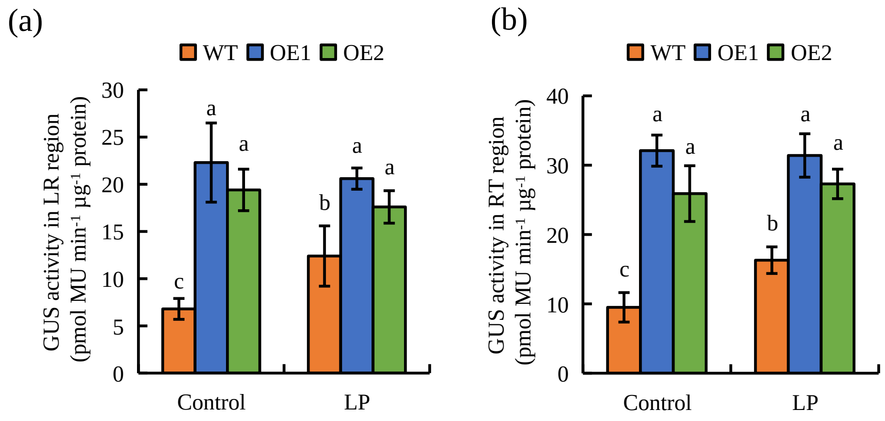

Figure S2. *DR5::GUS* enzyme activity of roots in wild type (WT, Nipponbare) and *OsPIN2* overexpression lines (OE1/OE2). Seedlings were grown in hydroponic media containing normal nutrition (control, 300 μM P) and low P (LP, 10 μM) for 7 d. *DR5::GUS* enzyme activity in lateral root (LR) region (**a**) and root tip (RT) region (**b**). d = days. Data are means ± SE and bars with different letters in the same root zone indicate significant difference at $p < 0.05$ tested with ANOVA.

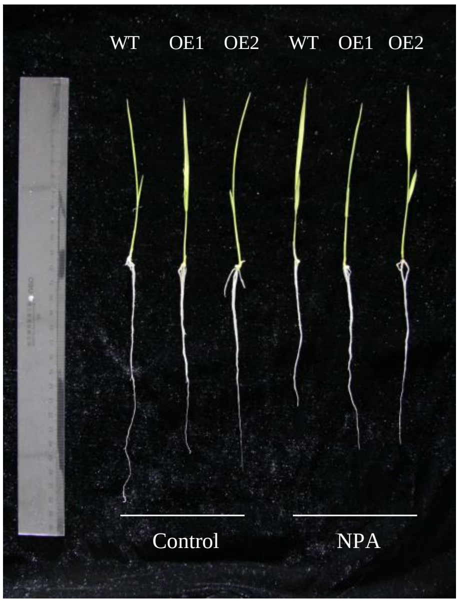

Figure S3. Root morphology in wild type (WT, Nipponbare) and *OsPIN2* overexpression lines (OE1/OE2). Seedlings were grown in hydroponic media containing normal nutrition (300 μM P) with or without NPA for 7 d. d = days.

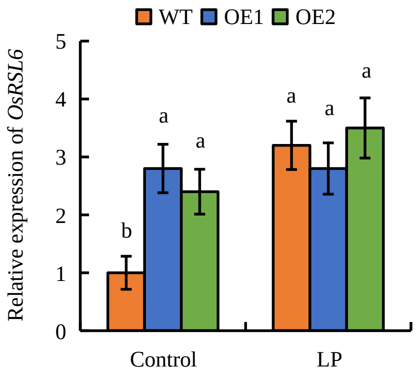

Figure S4. qRT-PCR analysis of *RSL6* gene in wild type (WT, Nipponbare) and *OsPIN2* overexpression lines (OE1/OE2). Seedlings were grown in hydroponic media containing normal nutrition (control, 300 μM P) and low P (LP, 10 μM) for 6 h. Relative mRNA levels were normalised to *OsACT*. h = hours. Data are means ± SE and bars with different letters indicate significant differences in the same gene ($p < 0.05$, ANOVA).
